# Pre-clinical dose-escalation studies establish a therapeutic range for U7snRNA-mediated *DMD* exon 2 skipping

**DOI:** 10.1016/j.omtm.2021.03.014

**Published:** 2021-03-23

**Authors:** Tabatha R. Simmons, Tatyana A. Vetter, Nianyuan Huang, Adeline Vulin-Chaffiol, Nicolas Wein, Kevin M. Flanigan

**Affiliations:** 1 Center for Gene Therapy, Abigail Wexner Research Institute, Nationwide Children’s Hospital, Columbus, OH 43205, USA; 2 Department of Pediatrics, Ohio State University, Columbus, OH, USA

**Keywords:** Duchenne muscular dystrophy, Becker muscular dystrophy, dystrophin, exon skipping, U7snRNA, gene therapy

## Abstract

Duchenne muscular dystrophy (DMD) is an X-linked progressive disease characterized by loss of dystrophin protein that typically results from truncating mutations in the DMD gene. Current exon-skipping therapies have sought to treat deletion mutations that abolish an open reading frame (ORF) by skipping an adjacent exon, in order to restore an ORF that allows translation of an internally deleted yet partially functional protein, as is seen with many patients with the milder Becker muscular dystrophy (BMD) phenotype. In contrast to that approach, skipping of one copy of a duplicated exon would be expected to result in a full-length transcript and production of a wild-type protein. We have developed an adeno-associated virus (AAV)-based U7snRNA exon-skipping approach directed toward exon 2, duplications of which represent 10% of all *DMD* duplication mutations. Deletion of exon 2 results in utilization of an exon 5 internal ribosome entry site (IRES) that allows translation beginning in exon 6 of a highly protective dystrophin protein, providing a wide therapeutic window for treatment. Both intramuscular and systemic administration of this vector in the Dup2 mouse model results in robust dystrophin expression and correction of muscle physiologic defects, allowing dose escalation to establish a putative minimal efficacious dose for a human clinical trial.

## Graphical abstract

**Figure:**
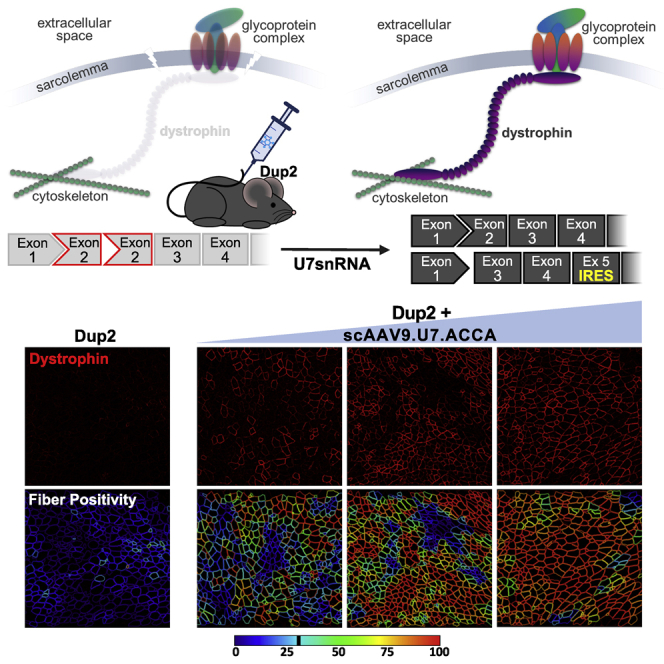

## Teaser

Flanigan and colleagues report the results of an intravenous dose-escalation study using an AAV9-encapsidated U7snRNA vector to skip *DMD* exon 2 and restore dystrophin expression in the Dup2 mouse model of Duchenne muscular dystrophy. The results informed dose considerations for an ongoing clinical trial.

## Introduction

The X-linked *DMD* gene, which encodes the dystrophin protein, consists of 79 exons and at least 7 promoters. Mutations in *DMD* result in either the severe Duchenne or milder Becker muscular dystrophy (DMD or BMD)—collectively, dystrophinopathies—with the predominant determinant of phenotype being whether or not the mutation maintains an open reading frame (ORF) that allows expression of a partially functional protein.^1^ Deletions of one or more exons constitute the most common mutation class in the dystrophinopathies, accounting for approximately 65% of all mutations.^2^^,^^3^ One therapeutic approach for these mutations has been exon skipping, in which pre-mRNA splicing is altered by the use of antisense oligomers directed to exons adjacent to those containing mutations; the resultant mRNA contains a larger deletion, but one in which the reading frame is restored.^4^, ^5^, ^6^

Exon duplications are less frequent, accounting for 6%–11% of all mutations in large dystrophinopathy cohort studies.^2^^,^^7^ The most common single exon duplicated is exon 2 (Dup2), accounting for around 10% of all *DMD* duplication mutations and thus around 0.6%–1.1% of all dystrophinopathy mutations. Duplications of exon 2 result in an altered reading frame, with a premature stop codon in the new reading frame within the exon 3 sequence. Consistent with this, the majority (80%) of Dup2 patients present with a typical DMD phenotype, with loss of ambulation before the age of 15 years. The remainder meet a common classification for BMD, with later loss of ambulation, although nearly all of these outliers have lost ambulation by the early 20s (unpublished data). In contrast, only one person has been described with a deletion of exon 2 (Δ2).^8^ Although Δ2 also results in a frameshift and premature stop codon in a new reading frame in the exon 3 sequence, this person was essentially asymptomatic, except for myalgias and elevated serum creatine kinase (CK). The difference between the Dup2 and the Δ2-associated phenotypes was explained by the identification of an internal ribosome entry site (IRES) within the *DMD* exon 5, which allows for cap-independent translational initiation in the case of an exon 2 deletion but is nonfunctional in the presence of an exon 2 duplication.^8^ Translation from this IRES results in the expression of a dystrophin isoform missing the calponin-homology 1 (CH1) domain within the N-terminal actin binding domain 1 (ABD1), but which is nevertheless highly functional; expression of the same isoform is found in patients across North America carrying the *DMD* p.Trp3X founder allele, resulting in mildly symptomatic BMD with ambulation into the seventh or eighth decade.^9^

As a result of these clinical observations, we may assume that skipping of exon 2 is a therapeutic approach that offers a very broad therapeutic window. Differing from the case with skipping for deletion mutations, which results in creation of a BMD-like internally truncated transcript, skipping a single copy of exon 2 would restore an entirely normal mRNA transcript. However, skipping of both copies of exon 2 results in a stable transcript subject to translation, via the exon 5 IRES, of a dystrophin isoform that is highly stable, as demonstrated by multiple patients with an exon 1 nonsense mutation who express the same isoform with symptoms of only myalgias and hyperCKemia.^9^ To test this as a potential therapeutic approach, we developed a mouse model with a duplication of exon 2 (Dup2).^10^ In this mouse dystrophin expression is essentially absent, and pathologic features and physiologic defects are essentially identical to the common *mdx* mouse model, which carries a nonsense mutation in exon 23 (rendering it unsuitable for testing exon 2 skipping therapy).

In contrast to exon skipping using a phosphorodiamidate morpholino oligomer (PMO),^6^^,^^11^ we designed modified U7snRNAs with terminal antisense oligonucleotide sequences that target exon 2 definition elements. The U7snRNAs, delivered in an adeno-associated virus (AAV) genome, are transcribed but never translated and are stable for long-term inducement of exon skipping.^12^, ^13^, ^14^, ^15^ We designed two such U7snRNAs, targeting either the exon 2 splice acceptor (sequence A) or splice donor (sequence C) sites, and cloned four copies (two of each sequence) into an AAV genome that we packaged within a self-complementary AAV9 vector (scAAV9.U7.ACCA) to demonstrate efficient skipping in human-derived cell culture and preliminary mouse studies.^8^

Here we present dose-escalation studies of both intramuscular (i.m.) and systemic delivery of scAAV9.U7.ACCA that show efficient skipping of exon 2, along with increased expression of properly localized dystrophin that restores muscle function. These data suggest that skipping of a duplicated exon 2 may be a feasible therapeutic approach, particularly because skipping of exon 2 may be associated with an apparently unlimited therapeutic window due to utilization of the IRES with complete exon 2 exclusion, and suggest that patients harboring other mutations 5′ of the exon 5 IRES may potentially benefit from expression of this highly functional dystrophin isoform via the same mechanism.

## Results

### i.m. dose escalation

**Figure 1:**
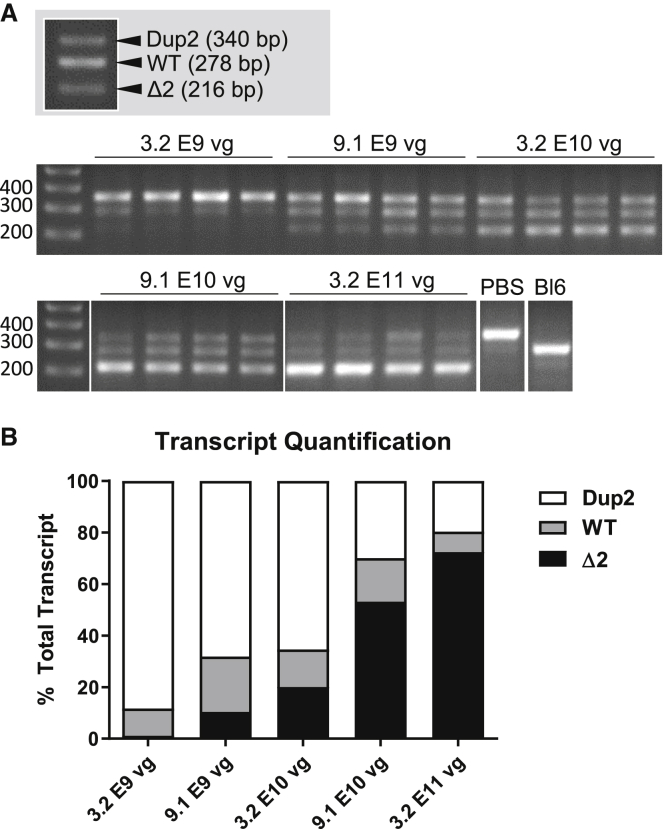
RT-PCR analysis following intramuscular (i.m.) injection of the tibialis anterior (TA) in Dup2 mice (A) Representative images of *DMD* RT-PCR show 3 different bands corresponding to the Dup2 (340 bp), wild-type (WT, 278 bp), and Δ2 (216 bp) transcripts. (B) Quantification of each transcript shows increasing efficiency of exon 2 skipping with scAAV9.U7.ACCA dose escalation, resulting in therapeutic transcripts (both WT and Δ2) 4–5 weeks post-injection. Quantification is reported as the mean % of total transcript for each transcript species in each dose group (n ≥ 8 per dose).

To determine if we could titrate skipping of exon 2 to a wild-type (WT) transcript, we performed an i.m. dose escalation in the tibialis anterior (TA) muscle by delivering 5 single doses at half-log increments. RT-PCR was used to evaluate the amount of exon 2 skipping seen at the mRNA level, where potential transcripts include a duplication of exon 2 (Dup2), a single copy of exon 2 (WT), or zero copies of exon 2 (Δ2) (Figure 1). The results show an expected dose response, with the lowest dose of 3.2 × 10^9^ vg resulting in 11% WT transcript, and the incremental dose of 9.1 × 10^9^ vg showing both the WT and Δ2 transcripts, each comprising at least 10% of the total transcript. At the highest dose of 3.2 × 10^11^ vg, only 20% of the transcript is Dup2, while the Δ2 transcript represents almost 75% of the total transcript (Figure 1).

**Figure 2:**
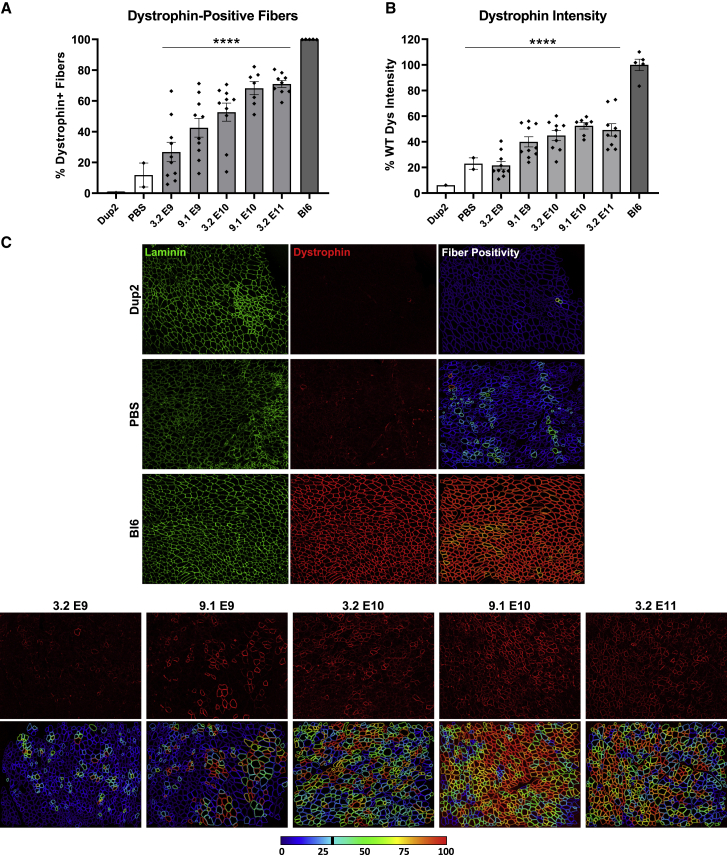
Dystrophin expression following i.m. injection of scAAV9.U7.ACCA in the TA in Dup2 mice (A and B) Immunofluorescence staining shows abundant and dose-dependent dystrophin expression 4–5 weeks after a single i.m. injection into the TA, quantified as dystrophin-positive fibers (A) and dystrophin signal intensity normalized to Bl6 muscle (B). Data presented as mean ± SEM, with individual data points representing individual samples. Statistical analysis was performed using one-way ANOVA with a post hoc test for linear trend from left to right; ∗∗∗∗p < 0.0001 for trend. (C) Representative images of TA sections confirm correct dystrophin localization (red) and depict quantification of fiber dystrophin positivity. Images were processed uniformly with automatic shading correction, rolling-ball background subtraction, and denoising as a part of preparation for quantitative analysis (see Figure S1 for unprocessed images). The color-coded heatmaps of each image reflect the percent of the perimeter with dystrophin-positive pixels for each muscle fiber identified using the laminin channel (green). Fibers that have dystrophin around ≥30% of the perimeter are considered dystrophin positive. The color scale indicates the conversion between color and % dystrophin-positive perimeter.

**Figure 3:**
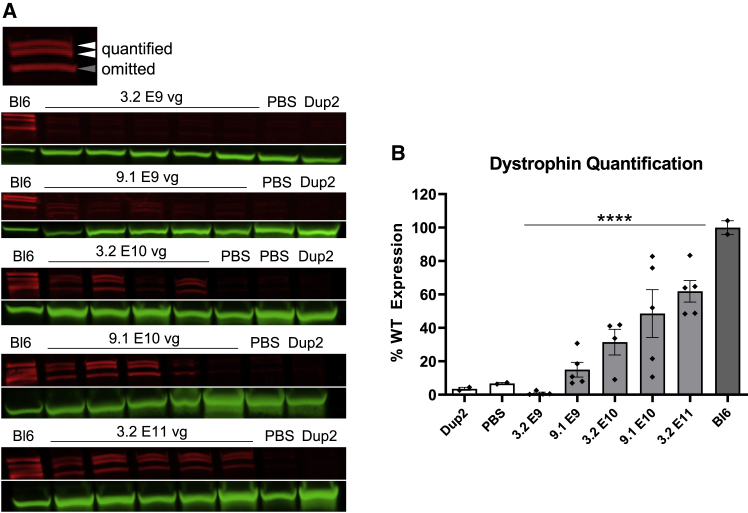
Immunoblot analysis following i.m. injection of scAAV9.U7.ACCA in the TA in Dup2 mice (A) Assessment of dystrophin expression by immunoblotting shows a dose-dependent increase in dystrophin protein that is confirmed by quantification of the dystrophin band doublet (red) normalized to actinin (green). (B) Quantification results reported as mean ± SEM, with individual data points representing individual samples. Statistical analysis was performed using one-way ANOVA with a post hoc test for linear trend from left to right; ∗∗∗∗p < 0.0001 for trend.

Dystrophin immunofluorescence (IF) analysis on frozen TA sections shows a similar progressive dose response for both dystrophin-positive fibers (Figure 2A) and dystrophin signal intensity (Figure 2B), confirmed by a significant linear trend from left to right. The mean proportion of dystrophin-positive fibers ranged from 27% up to 71% across all i.m. dose groups in contrast to <20% in all untreated Dup2 sections, and dystrophin signal intensity peaked around 52% of median WT intensity at the sarcolemma. Representative images confirm proper sarcolemmal localization at all 5 doses tested (Figure 2C) and demonstrate sparse sarcolemmal dystrophin staining at low doses that increases to include the vast majority of fibers at higher doses, consistent with the RT-PCR analysis. Dystrophin protein was further quantified by immunoblot (IB), confirming the progressive dose response and showing 62% WT dystrophin levels at the highest dose of 3.2 × 10^11^ vg (Figure 3).

**Figure 4:**
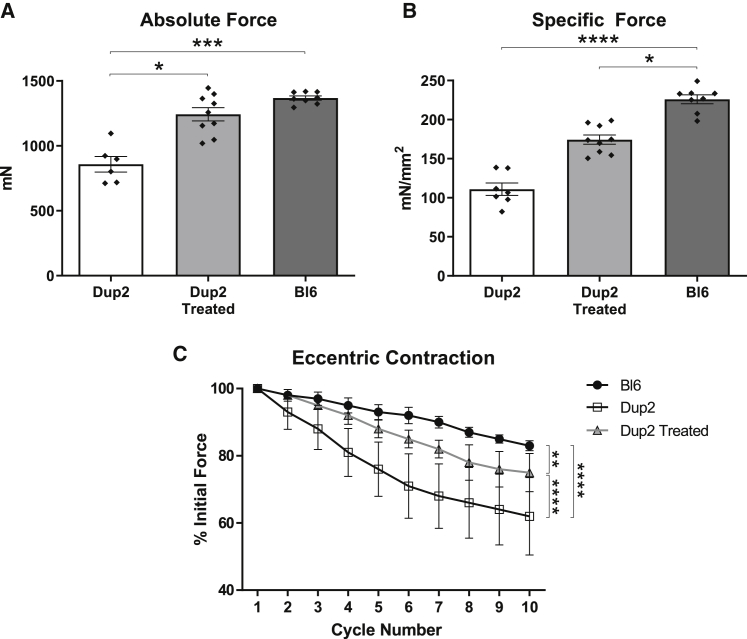
Functional correction following i.m. injection of scAAV9.U7.ACCA (A and B) Injection of scAAV9.U7.ACCA at the maximal i.m. dose is associated with a near-complete rescue of absolute force (A) and a partial rescue of specific force (B) 4–5 weeks after treatment. (C) Similar to specific force, force drop following repeated eccentric contractions shows a partial rescue with treatment. All results presented as mean ± SEM, with individual points reflecting individual muscles in absolute and specific force experiments. Absolute and specific force data were analyzed using a Kruskal-Wallis test with Dunn’s multiple comparison test for pairwise comparisons, and eccentric contraction force drop was analyzed by two-way ANOVA with the Holm-Sidak multiple comparison test; ∗p < 0.05; ∗∗p < 0.01; ∗∗∗p < 0.001; ∗∗∗∗p < 0.0001.

To determine the degree of functional rescue, *in vivo* muscle function studies were performed on additional TA muscles 4 weeks after a single i.m. injection of 3.2 × 10^11^ vg, measuring both force (absolute and specific) and force drop following repeated eccentric contractions. Treated Dup2 muscle showed 45% higher mean absolute force (Figure 4A) and 64% higher specific force (Figure 4B) relative to untreated Dup2, although the dataset lacked sufficient statistical power for the difference in specific force to reach statistical significance. Despite the near-complete correction of absolute force, specific force in treated Dup2 muscles remained significantly different from Bl6. The Dup2 mouse is generally larger than control Bl6 mice,^10^ and this difference in size may account for the discrepancy between recovery of the absolute and specific force, as specific force takes into account muscle cross-sectional area. Similarly, treated Dup2 muscles show a significant partial rescue of force drop following repeated eccentric contractions but remain more sensitive to this injury than Bl6 muscles (Figure 4C).

### Intravenous dose escalation

**Figure 5:**
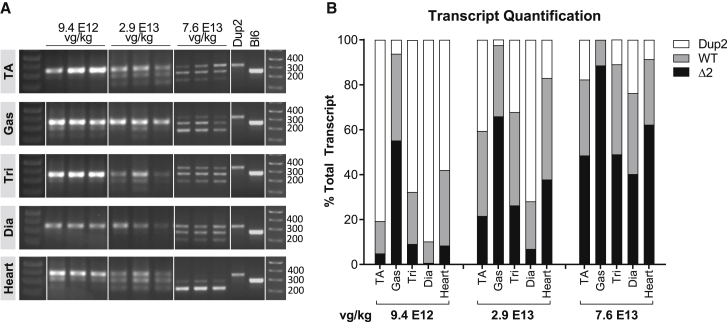
Systemic delivery of scAAV9.U7.ACCA results in significant exon skipping at 4 weeks post-injection (A) Tissues from Dup2 mice treated at 8–9 weeks of age were assessed 4 weeks post-injection by RT-PCR for relative amounts of *DMD* transcripts having 2 (Dup2, 340 bp), 1 (WT, 278 bp), or 0 (Δ2, 216 bp) copies of exon 2. (B) Exon 2 skipping is clearly present, and quantification demonstrates a dose response at the 3 highest doses shown here. Quantification is reported as the mean % of total transcript for each transcript species in muscle tissue representing the 3 highest doses (n = 3 per tissue and dose).

With an ultimate goal of systemic delivery to reach all skeletal muscles, heart, and diaphragm, we next undertook systemic intravenous (i.v.) dose-escalation studies in order to establish a minimal efficacious dose (MED) necessary for designing IND-enabling toxicology studies, as well as for eventual clinical translation. We initially performed single tail vein injections of 6 ascending doses and analyzed outcomes 4 weeks later. The results of RT-PCR analysis (Figure 5) show that the Dup2 transcript was the major species at the lowest doses (2.9 × 10^11^ vector genomes per kilogram (vg/kg) to 2.9 × 10^12^ vg/kg), accounting for at least 60% of transcripts. Higher doses, ranging from 9.4 × 10^12^ vg/kg to 7.6 × 10^13^ vg/kg, were associated with increased skipping of either one or two copies of exon 2. At the highest dose of 7.6 × 10^13^ vg/kg, skipped transcripts represented 42%–96% of total transcripts in different muscle types. Notably, both the WT and the Δ2 transcripts are therapeutic, given the presence of the dystrophin exon 5 IRES.

**Figure 6:**
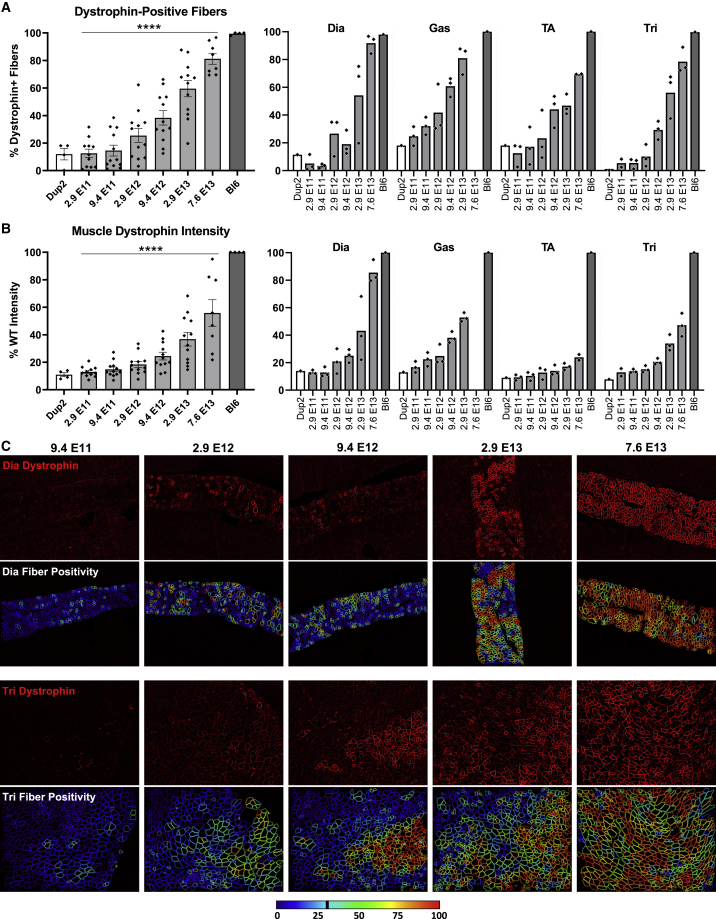
Systemic delivery of scAAV9.U7.ACCA results in significant dystrophin expression in skeletal muscle 4 weeks post-injection (A and B) Immunofluorescence staining confirmed dystrophin expression in four representative skeletal muscles 4 weeks after an i.v. injection, quantified as dystrophin-positive fibers (A) and dystrophin signal intensity normalized to Bl6 signal (B). Data presented as mean ± SEM, with individual data points representing individual samples. Statistical analysis was performed on pooled data from all skeletal muscles using one-way ANOVA with a post hoc test for linear trend from left to right; ∗∗∗∗p < 0.0001 for trend. Right panel of (A) and (B) displays the quantification results broken down by each muscle. (C) Representative images of diaphragm (Dia) and triceps (Tri) sections from the 5 highest dose groups confirm correct dystrophin localization (red) and display quantification of fiber dystrophin positivity. Images were processed uniformly with automatic shading correction, rolling-ball background subtraction, and denoising as a part of preparation for quantitative analysis (see Figure S2A for unprocessed images). The color-coded heatmaps of each image reflect the percent of the fiber perimeter with dystrophin-positive pixels. Fibers that have dystrophin around ≥30% of the perimeter are considered dystrophin positive. The color scale indicates the conversion between color and % dystrophin-positive perimeter.

**Figure 7:**
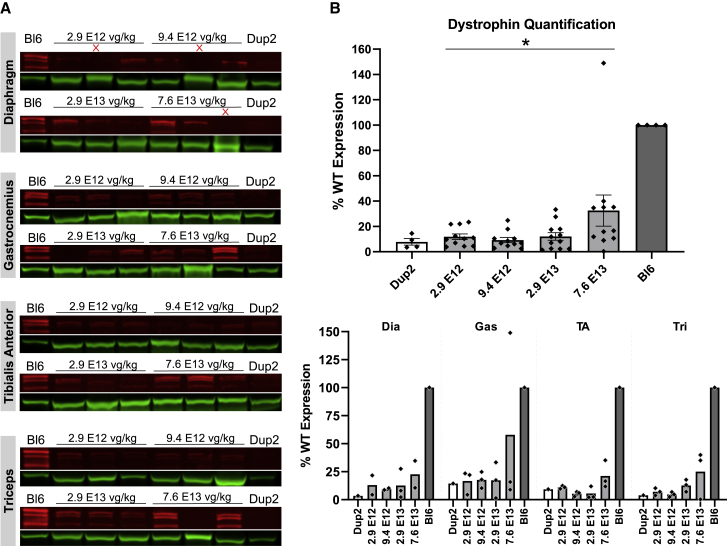
Immunoblot analysis following delivery of scAAV9.U7.ACCA confirms dystrophin expression 4 weeks post-injection (A and B) Representative images of immunoblots (A) show increase in dystrophin protein (red) normalized to actinin (green), and quantification (B) confirms a significant increase in dystrophin protein expression with increasing dose. Sample lanes marked with a red X were omitted due to technical issues. Quantification reported as mean ± SEM, with individual data points representing individual samples. Statistical analysis was performed on pooled data from all skeletal muscles using one-way ANOVA with a post hoc test for linear trend from left to right; ∗p < 0.05 for trend. Lower panel displays quantification results broken down by individual muscles.

IF images and analysis (Figure 6) are consistent with RT-PCR results, showing modest increases in dystrophin-positive fibers and dystrophin signal intensity at lower doses and robust restoration of properly localized dystrophin at higher doses across all skeletal muscles. At the highest dose of 7.6 × 10^13^ vg/kg, dystrophin is present in 69%–92% of fibers on average across different skeletal muscles, with a global mean of 81% for pooled data from all skeletal muscles (Figure 6A). Dystrophin signal intensity restoration at the sarcolemma was less robust, with a global mean of 56% of Bl6 intensity for pooled data from all skeletal muscles at highest dose, which is 4.1-fold higher than untreated Dup2 intensity (Figure 6B). Quantification of dystrophin expression by immunoblot confirms a dose-related increase in dystrophin protein, reaching a global mean of 34% of Bl6 expression, 4.4-fold higher than untreated Dup2, at the highest dose of 7.6 × 10^13^ vg/kg (Figure 7).

**Figure 8:**
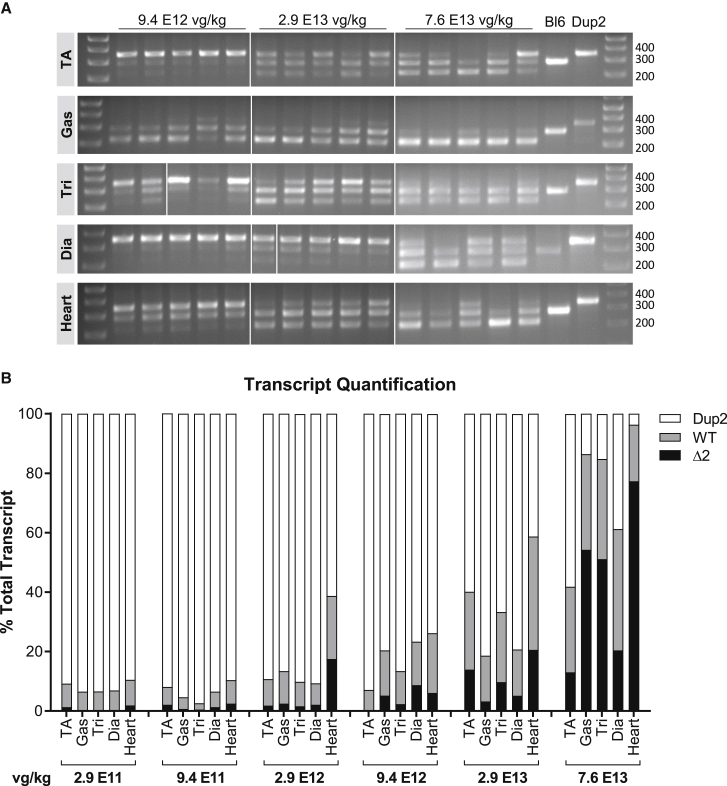
Expression of therapeutic *DMD* transcripts is evident 12 weeks after systemic scAAV9.U7.ACCA injection (A) Exon 2 skipping was assessed by RT-PCR 12 weeks post-injection in muscle tissues by visualization of amplicons representing *DMD* transcripts having 2 (Dup2, 340 bp), 1 (WT, 278 bp), or 0 (Δ2, 216 bp) copies of exon 2. (B) RT-PCR quantification demonstrates that the proportion of therapeutic transcripts (WT or Δ2) relative to the total transcript increases by dose. Quantification is reported as the mean % of total transcript for each transcript species in muscle tissue representing the 6 treatment doses (n = 5–6 per tissue and dose).

**Figure 9:**
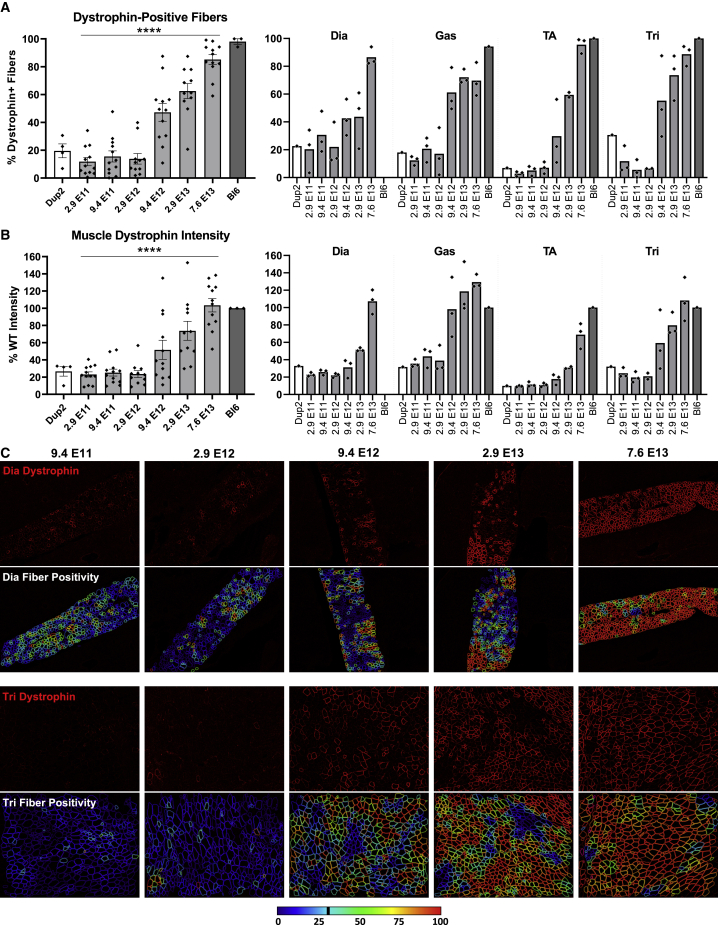
I.v. delivery of scAAV9.U7.ACCA results in dose-dependent expression of dystrophin protein at 12 weeks post-injection (A and B) Dystrophin immunofluorescence confirms sustained dystrophin expression 12 weeks after systemic injection of scAAV9.U7.ACCA in four representative skeletal muscles, as shown by dystrophin-positive fibers (A) and dystrophin signal intensity normalized to Bl6 signal (B). Diaphragm Bl6 tissue images collected with identical exposure settings were not available for analysis, so diaphragm intensities are normalized to the mean of dystrophin intensities in the other three Bl6 skeletal muscles. Data presented as mean ± SEM, with individual data points representing individual samples. Statistical analysis was performed on pooled data from all skeletal muscles using one-way ANOVA with a post hoc test for linear trend from left to right; ∗∗∗∗p < 0.0001 for trend. Right panel of (A) and (B) displays the quantification results broken down by each muscle. (C) Representative images of diaphragm (Dia) and triceps (Tri) sections from the 5 highest dose groups confirm correct dystrophin localization (red) and reflect fiber dystrophin positivity. Images were processed uniformly with automatic shading correction, rolling-ball background subtraction, and denoising as a part of preparation for quantitative analysis (see Figure S2B for unprocessed images). The color-coded heatmaps of each image reflect the percent of the fiber perimeter with dystrophin-positive pixels. Fibers that have dystrophin around ≥30% of the perimeter are considered dystrophin positive. The color scale indicates the conversion between color and % dystrophin-positive perimeter.

**Figure 10:**
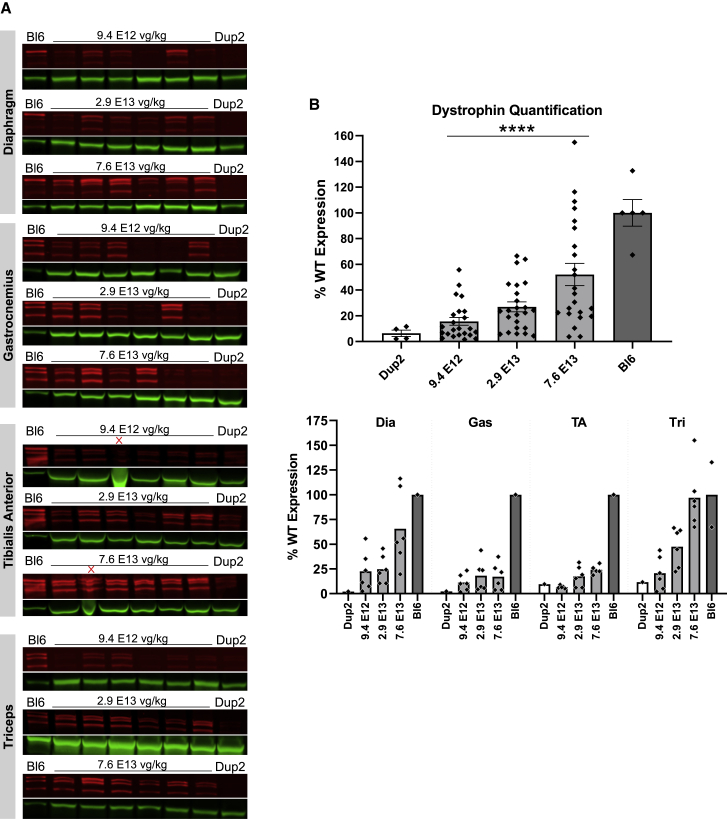
Immunoblot analysis demonstrates continued dystrophin expression 12 weeks after i.v. injection of scAAV9.U7.ACCA (A) Immunoblots for dystrophin show a sustained increase in dystrophin protein expression (red) normalized to actinin (green) across four different muscles 23 weeks after treatment. Sample lanes marked with a red X were omitted due to technical issues. (B) Immunoblot quantification confirms a significant increase in dystrophin protein expression with increasing dose. Quantification reported as mean ± 95% CI (shaded region), with individual data points representing individual samples. Quantification reported as mean ± SEM, with individual data points representing individual samples. Statistical analysis was performed on pooled data from all skeletal muscles using one-way ANOVA with a post hoc test for linear trend from left to right; ∗∗∗∗p < 0.0001 for trend. Bottom panel displays quantification results broken down by individual muscles.

In order to further define a clinically relevant minimal efficacious dose, and to assess the longevity of dystrophin expression, we injected another cohort of Dup2 mice with the same set of 6 i.v. doses and analyzed dystrophin expression and muscle function 12 weeks post-injection. With the exception of IF, only results for the 3 highest doses are shown due to a negligible effect at the 3 lower doses. RT-PCR analysis (Figure 8) shows increased exon 2 skipping in all doses, with single or double skipping of exon 2 representing at least 75% of the total transcript at the highest dose of 7.6 × 10^13^ vg/kg. IF shows a marked increase in dystrophin expression at the 3 highest doses, with near complete average restoration of dystrophin-positive fibers (85%) and complete restoration of mean sarcolemmal dystrophin intensity (104% of Bl6 intensity) at the highest dose in all muscles (Figure 9). Immunoblot analysis corroborates progressively increasing dystrophin levels at the 3 upper doses, with mean dystrophin content of 52% of Bl6 at the highest dose of 7.6 × 10^13^ vg/kg (Figure 10).

**Figure 11:**
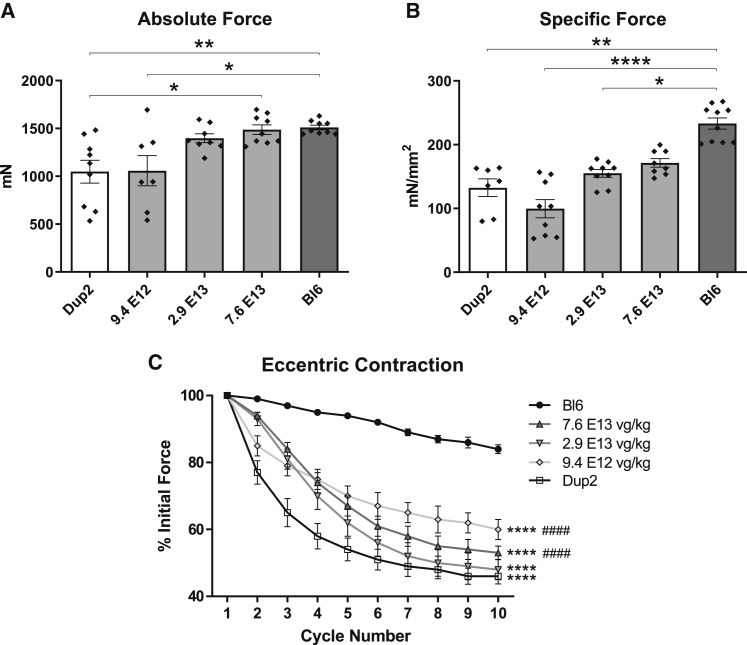
Correction of force and protection from muscle injury 12 weeks after systemic delivery of sc.AAV9.U7.ACCA (A and B) Muscle function testing performed on 20- to 21-week-old mice 12 weeks after treatment shows a dose-dependent response, with a complete rescue of absolute force (A) at the two highest doses and a partial rescue of specific force (B) at the highest dose. All results presented as mean ± SEM, with individual points reflecting individual muscles. Absolute and specific force data were analyzed using a Kruskal-Wallis test with Dunn’s multiple comparison test for pairwise comparisons; ∗p < 0.05; ∗∗p < 0.01; ∗∗∗∗p < 0.0001. (C) Force drop following repeated eccentric contractions also shows a partial improvement after scAAV9.U7.ACCA treatment. Eccentric contraction-induced force drop is presented as mean ± SEM and was analyzed by two-way ANOVA with the Holm-Sidak multiple comparison test; ∗∗∗∗p < 0.0001 versus Bl6; ^####^p < 0.0001 versus Dup2.

Functional studies performed on the TA muscle of treated Dup2 mice 12 weeks after injection also showed progressively increasing absolute and specific force at the 3 highest doses, culminating in complete rescue of absolute force (Figure 11A) and partial rescue of specific force (Figure 11B). Muscle force after multiple eccentric contractions again showed a partial rescue, with intermediate force drop observed in treated Dup2 that was significantly different from both untreated Dup2 and Bl6 muscles (Figure 11C).

**Figure 12:**
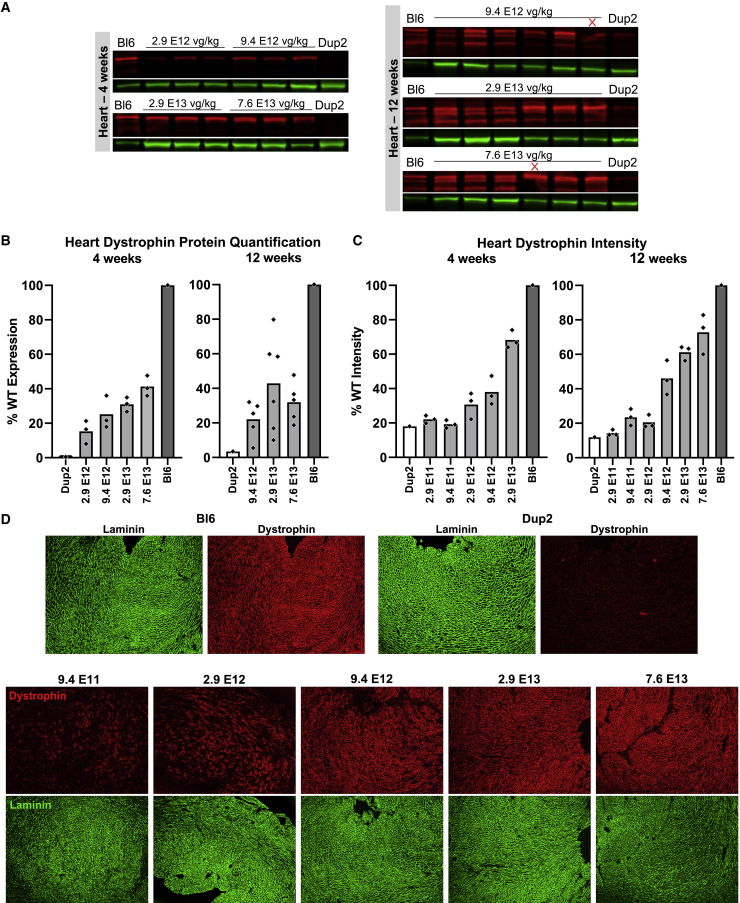
I.v. delivery of scAAV9.U7.ACCA induces abundant dystrophin expression in the heart (A and B) Immunoblotting (A) for dystrophin (red) in the heart and subsequent quantification (B) shows successful restoration of dystrophin expression 4 weeks and 12 weeks after i.v. treatment with scAAV9.U7.ACCA. Sample lanes marked with a red X were omitted due to technical issues. (C) Quantification of dystrophin immunofluorescence intensity in the heart further confirms dose-dependent dystrophin restoration 4 weeks and 12 weeks after treatment. Cardiac dystrophin levels were measured under a laminin mask as mean dystrophin intensity normalized to mean laminin intensity and expressed as a percentage of Bl6. Immunoblot and immunofluorescence quantification data plotted as the mean with individual data points representing individual hearts. Due to a particularly small number of samples, statistical analysis was not performed. Representative images of hearts collected 12 weeks post-treatment (C) demonstrate the levels of dystrophin signal (red) and laminin signal (green) in hearts from Dup2 and Bl6 controls and the 5 highest treatment dose groups. Images were processed uniformly with automatic shading correction, rolling-ball background subtraction, and denoising as a part of preparation for quantitative analysis (see Figure S3 for unprocessed images).

Due to the prevalence of cardiomyopathy among patients with DMD, successful dystrophin restoration in the heart is an important aspect of the potential clinical benefits of gene therapies. Immunoblot and IF results reflect effective dystrophin expression in treated Dup2 hearts at time points of 4 weeks and 12 weeks post-injection (Figure 12). Immunoblots showed dystrophin levels reaching just over 40% at the higher doses at both time points (Figure 12B), and average dystrophin signal intensity at the sarcolemma reached 73% of Bl6 levels (5.2-fold higher than untreated Dup2) at the highest dose of 7.6 × 10^13^ vg/kg (Figure 12C).

## Discussion

Systemic delivery of scAAV9.U7.ACCA induces robust skipping of exon 2 within *DMD* mRNA in skeletal muscle, heart, and diaphragm of Dup2 mice. This skipping results in two transcripts, consisting of the WT mRNA and an mRNA in which no copies of exon 2 are included (the Δ2 transcript). Both would be therapeutic, because translation of the Δ2 mRNA (via utilization of the *DMD* IRES) results in a highly functional isoform of approximately 413 kDa, which—despite a partial N-terminal deletion—is associated with either a very mild BMD or an essentially asymptomatic state.

The fact that the IRES is utilized in the absence of exon 2 makes the prospect of therapeutic exon 2 skipping particularly attractive due to the large therapeutic window, as “overskipping” results in the therapeutic Δ2 transcript. This is in contrast to out-of-frame duplications of exons within the central rod mutational hotspot of *DMD* that are typically associated with DMD, as predicted by the reading frame rule. In that region, only the single exon copy WT transcript would be therapeutic, while total exclusion of the exon would be expected to result in an out-of-frame deletion transcript that cannot be translated. Clinically, a DMD patient with a duplication in this region would not be expected to be made by overskipping, as both the exon-duplicated and deleted transcripts truncate the reading frame. However, the therapeutic window leading to induction of a WT transcript and optimal dystrophin expression response might be narrow. We note that in the present study we were unable to titrate dosage to a response consisting of only the WT transcript, suggesting a high efficiency for U7snRNA-mediated exon 2 skipping, but skipping efficiency of other exons may vary, and titration to single-copy skipping may be feasible.

Exon 2 skipping as measured by total therapeutic transcripts corresponds with a dose-related increase in levels of dystrophin, as quantified by immunoblotting and assessed qualitatively by IF, which also shows proper localization of dystrophin. The inference from the mRNA studies is that the protein recognized by the C-terminally directed antibody is expected to be both WT and N-deleted isoforms, and it is unfortunate that the only antibody that might distinguish the presence of exon 1-specific epitopes translated from the WT mRNA is a mouse monoclonal (Manex1A) that has proven inadequately sensitive to distinguish differential expression on mouse frozen sections. Regardless of the isoform expressed, systemic delivery of scAAV9.U7.ACCA results in complete restoration of absolute force, as well as a partial rescue in both specific force and force drop following repeated eccentric contractions, at least in the TA muscle—another unsurprising finding, given the protection that expression of the N-deleted isoform confers on patients who express it as a founder allele.^9^

Although RT-PCR and dystrophin expression following i.m. injection may be useful measures of potency for comparing vectors or vector lots, successful systemic delivery will be critical to therapeutic translation, given that muscle represents 30%–40% of adult body mass.^16^ Transduction and dystrophin correction in the heart and diaphragm are critical, as most DMD patients die of cardiac or respiratory insufficiencies. We note some variability between skeletal muscles in expression, which may represent differences in regional perfusion or even in vector tropism, but it is promising that in our dose-escalation series, expression of dystrophin in the heart in particular seemed even more robust than in skeletal muscle at comparable doses. Comparison of the 12-week versus 4-week post-injection studies shows that both exon skipping and dystrophin expression are more robust at the later time point. We interpret this to mean that as the expression of dystrophin protein occurs, the stability of the resultant WT and N-deleted isoforms results in their continued accumulation at the sarcolemmal membrane at longer time points, and expression at much longer time points is the subject of another report in preparation.^8^

We are reluctant to compare too directly the degree of this protection from eccentric contraction damage in our hands to studies of other dystrophins from other labs for several reasons. First, it is our experience (and, we believe, the experience of many in the field) that electrophysiologic studies show marked variability, even among control untreated *mdx* mice, among multiple labs (as shown in the range of published results). Second, the Dup2 mouse and the classic *mdx* mouse are on different backgrounds. Third, the clinical findings in humans are a stronger argument for the functionality of the IRES-driven protein than any physiologic modeling in the mice. This isoform, which lacks the calponin-homology 1 domain and is translated from the exon 5 IRES, was first identified in a pedigree in which the proband was ambulant into his seventh decade and whose brother was essentially asymptomatic at age 58.^17^ It was subsequently defined as the isoform expressed by all subjects studied in characterizing the North American *DMD* p.Trp3X founder allele, who had hyperCKemia, myalgias, and cramps but no significant weakness.^9^ The IRES utilization occurs with many 5′ mutations, and, consistent with prior findings in the papers above, the first male identified with a deletion of exon 2 expressed the same isoform and had no significant weakness; in the same publication, expression of this IRES-driven isoform in mice resulted in restoration of membrane integrity and muscle force.^8^ In short, there can be no doubt that this is a highly functional protein, and these clinical observations provide much better evidence for the functionality of the protein itself than exists, for example, for engineered microdystrophin, for which there are no exact existing clinical correlates.

In summary, we believe our results suggest that systemic delivery of scAAV9.U7.ACCA is feasible and that a minimal efficacious dose of 3 × 10^13^ vg/kg results in robust expression of full-length or near-full-length dystrophin isoforms across skeletal muscles and the heart. We note that our data do not define a maximal efficacious dose, nor do they establish a dose range limited by toxicity. Nevertheless, these data provide a solid foundation for the design of a clinical dosing regimen and of the necessary IND-enabling toxicity studies.

## Materials and methods

### U7snRNA vector design and production

Vector for this study was produced in the Viral Vector Core at The Research Institute at Nationwide Children’s Hospital. Serotype 9 recombinant (rAAV) vectors were produced by a modified cross-packaging approach using an adenovirus-free, triple-plasmid DNA transfection (CaPO_4_ precipitation) method in human embryonic kidney 293 cells.^18^ Four independent U7snRNA cassettes containing a copy of an antisense sequence to either the splice acceptor (sequence A) or splice donor (sequence C) site were packaged into a scAAV2/9 (scAAV9.U7.ACCA) according to previously published methods.^8^ The production plasmids were: (1) scAAV9.U7.ACCA, (2) rep2-cap9 AAV helper plasmids encoding cap serotype 9, and (3) an adenovirus type 5 helper plasmid (pAdhelper) expressing adenovirus E2A, E4 ORF6, and VA I/II RNA genes. Physical titer determination was based on degradation of non-encapsidated DNA following digestion of viral capsids. Released encapsidated DNA was quantified by qPCR using a linear standard to determine the DNAse resistant particle (DRP) titer. (We note that the doses reported herein differ from those reported in the thesis of T.S. [unpublished data], deposited in the library of the Ohio State University. This reflects changes in the titering methodology used over the course of vector development. The titers we report here utilize the methodology used in subsequent IND-enabling studies.)

### Animal studies

Stocks of Dup2 and C57BL/6 (Bl6) mice were bred and maintained in standardized conditions in the vivarium at Nationwide Children’s Research Institute (IACUC protocol #AR10-00002 and IBCSC protocol #IBS00000201). Mice were kept under a 12:12 h dark:light cycle with free access to a low-fat diet and water. Due to the X-linked inheritance pattern of DMD, only male mice were used in these studies.

For the i.m. injection studies, 8-week-old Dup2 mice were injected into the TA with 5 doses of scAAV9.U7.ACCA between 3.2 × 10^9^ and 3.2 × 10^11^ total vector genomes (n = 10 limbs/dose for molecular studies) and sacrificed 4 weeks later. At sacrifice, TA, gastrocnemius, triceps, diaphragm, and heart were removed and frozen in liquid-nitrogen-cooled isopentane.

For the i.v. studies, an initial dose-escalation study was conducted wherein Dup2 mice were systemically injected via the tail vein at 8 weeks of age with 6 doses of scAAV9.U7.ACCA between 2.9 × 10^11^ vg/kg and 7.6 × 10^13^ vg/kg (N ≥ 3 per dose). Mice were sacrificed 4 weeks post-injection, and TA, gastrocnemius, triceps, diaphragm, and heart were removed and frozen in liquid-nitrogen-cooled isopentane. Increasing treatment time to 12 weeks, a second dose-escalation study was conducted following a single systemic injection into the tail vein of 8-week-old Dup2 mice with 6 doses of scAAV9.U7.ACCA between 9.4 × 10^12^ vg/kg and 7.6 × 10^13^ vg/kg (N ≥ 3 per dose).

### mRNA analysis

RNA was isolated from frozen muscle sections (15 × 40 μm sections) using Trizol according to the manufacturer’s protocol (Life Technologies, 15596018). For each sample, 1 μg of total RNA was used to generate cDNA according to the manufacturer’s protocol (Thermo Scientific, Ferk1672) and then used for a single PCR of 35 cycles. The primers used included a forward primer to exon 1 (5′-TACCTAAGCCTCCTGGAGCA-3′) and a reverse primer to the junction of exons 3 and 4 (5′-CTTTTGGCAGTTTTTGCCCTGTA-3′). The possible transcripts included a duplicated exon 2 (340 bp), WT with a single copy of exon 2 (278 bp), and a Δ2 transcript that had zero copies of exon 2 (216 bp). The PCR products were electrophoresed on a 2% agarose gel and imaged using a Gel Logic 200 Imaging System (Kodak). The images were then used to quantify the relative amount of each transcript using ImageJ software.^19^ Using the “Gel” function of ImageJ, a box was drawn in each lane around the area where the three possible transcripts would be. A histogram of each sample was then generated, and the area under the curve was measured. The sum of the area under the three peaks was added together, and then individual peak area was measured as a percentage of the total.

### IF and microscopy

Sections (10 μm) of frozen muscle were permeabilized (PBS, 2% Normal goat serum [NGS] and 0.1% Triton-X) for 10 min and then blocked with 15% NGS in PBS for 1 h. Primary antibody staining was performed for 2 h at room temperature or overnight at 4°C using a rabbit polyclonal anti-dystrophin (1:400, Abcam, ab15277) and a rat monoclonal anti-laminin (1:400, R&D Systems, MAB4656). Secondary antibody staining was performed for 1 h using Alexa Fluor 568 goat anti-rabbit (1:500, Invitrogen, A21069) and Alexa Fluor 488 donkey anti-rat (1:500, Jackson, 712-456-153) antibodies. Slides were then mounted using a 2.5% polyvinyl alcohol/1,4 diazabicyclo[2.2.2]octane solution.

Images of representative tissue regions were captured on an Olympus Bx61 motorized epifluorescence microscope using a DP71 camera with a 0.5× relay lens and a UPlanSApo 10× objective at a resolution of 1.28 μm/pixel.

### IF analysis

Nikon NIS-Elements AR software was used to perform automated analysis of dystrophin-positive fibers and dystrophin intensity in multichannel images of dystrophin and laminin IF. To maintain consistency, only image sets that were acquired within the same batch and under the same exposure settings were quantified together and compared, resulting in the exclusion of a small number of samples from quantification. In preparation for analysis, all images were preprocessed using automatic shading correction, rolling-ball background subtraction, and denoising to eliminate the impact of uneven background signal and illumination. The preprocessing steps were applied in an automated and identical fashion to all images.

For dystrophin-positive fiber analysis, the artificial intelligence (AI) NIS-Elements software module Segment.ai was trained to recognize muscle fibers outlined by laminin signal. The AI was trained using a set of 22 images of skeletal muscles spanning various treatment groups that each had a hand-drawn “ground truth” binary layer that showed all identifiable muscle fibers. AI training for 1,000 iterations with this image set spanned 7.5 h and resulted in a training loss of 0.018, roughly representing an error rate of ~1.8% relative to the hand-drawn ground truth in the training set. All laminin channel images were then processed through segmentation by the trained Segment.ai and checked for quality of fiber identification. Any images that showed inappropriate fiber segmentation due to poor tissue/staining quality or oblique fiber orientation were excluded from subsequent analysis.

Following segmentation, dystrophin-positive fibers were analyzed using thresholds for dystrophin-positive and laminin-positive pixels that were objectively derived based on the intensity of the signals in each image. Laminin-positive pixels in each image were identified by automatic thresholding using a multi-level Otsu algorithm. A single dystrophin-positive pixel threshold was calculated for each image set by measuring the 99.8^th^ percentile signal intensity in the non-sarcolemmal regions within each image, multiplying it by 1.5, and calculating the mean for all images within the set. Dystrophin-positive fibers were then identified by measuring the combined length of dystrophin-positive segments around each muscle fiber perimeter and normalizing it to the length of the laminin-positive perimeter around the same muscle fiber. A color-coded overlay image of all muscle fiber perimeter regions where measurements were collected was automatically exported as an output of the analysis. A muscle fiber was considered overall positive for dystrophin if 30% or more of the perimeter had dystrophin-positive signal. When multiple non-overlapping images of regions from the same tissue section were available, the total and positive fiber counts were pooled across the multiple images. Dystrophin signal intensity was measured under a laminin-positive image mask, and the dystrophin intensity was normalized to the laminin intensity within this mask for each image. Laminin-positive pixels were identified using an automatic multi-level Otsu threshold, and the mean intensity of these pixels was measured in both channels. When images of multiple non-overlapping regions of the same tissue section were available, the dystrophin:laminin intensity ratio for the tissue was expressed as a mean weighted in proportion to total laminin-positive mask area.

### Immunoblot analysis

Protein extractions were conducted starting with 25 sections (40 μm each) and 100 μL of lysis buffer containing a base buffer, a phosphatase inhibitor (PhosStop, Roche, 4906845001), and a protease inhibitor (Halt Protease Inhibitor Cocktail, Fisher, 78430). Steel beads were added to the tissue, which was homogenized using the Tissuelyser II (QIAGEN) for 2 min at a rate of 30/s. Lysates were then incubated on ice and spun down, the supernatant was removed and stored at −80°C until immunoblotting, and the cell debris was discarded. For immunoblotting, 50 μg of C57BL/6 or 150 μg of Dup2 protein was loaded with a loading dye (1× Laemmli) on a 3%–8% Tris acetate gel (Life Technologies, EA0378BOX). The gels were run for 30 min at 80V, and then for 4 h at 120 V. Protein was transferred to a nitrocellulose membrane (Fisher, 09-301-108) overnight at 4°C in transfer buffer (Invitrogen, NP00061) at 50 V. The membranes were then labeled using a rabbit polyclonal anti-dystrophin (1:200, Abcam, ab15277) and mouse monoclonal α-actinin (1:5,000, Fisher, MA122863) primary antibodies, followed by IRDye anti-rabbit 680 and anti-mouse 800 (Li-Cor, 926-68071 and 926-32210) secondary antibodies. The membrane was scanned on the Odyssey CLx and imaged using Image Studio.^19^ Single-channel images were obtained using Image Studio and then imported into ImageJ for quantification. For each channel, a box was drawn in each lane around the upper doublet using the Gel function in ImageJ (as demonstrated in Figure 3), as this encompasses both WT and IRES-driven protein. A line profile of each sample was then generated and the area under the curve corresponding to the dystrophin band doublet was measured. Dystrophin was normalized to α-actinin by dividing the area under the curve of the dystrophin band(s) by the area of the α-actinin band for each sample. If the same sample was run on multiple immunoblots, the median was taken to represent that particular sample. The C57BL/6 measurement was determined for each muscle by taking the mean of 2–3 technical replicates from separate blots that also included all treated and untreated control Dup2 samples for the given muscle. Both treated Dup2 and control Dup2 measurements are reported as a percentage of the WT. A small number of lanes showing unusually low overall intensities indicative of a failed transfer and bands with significant distortion were omitted from analysis.

### Force generation and protection from eccentric contractions

Physiologic studies were conducted on TA muscles from 12-week-old mice that had been treated with an i.m. injection of 3.2 × 10^11^ vg at 8 weeks (N ≥ 5 limbs). For systemic studies, the mice were 20–21 weeks old following 12 weeks of treatment (N ≥ 12 limbs). The force study procedure was conducted using a modified version of Hakim et al.^8^^,^^20^ Initial anesthetization of mice was conducted by giving an intraperitoneal injection of a cocktail contain five times their weight of 25 mg/mL ketamine and twice their weight of 2.5 mg/mL xylazine. The skin fascia and connective tissue were removed from around the TA. Throughout the procedure, the TA muscle was constantly moistened with 0.9% saline. A knot was tied to the distal TA tendon with a 4-0 suture and the tendon was cut. The excess suture thread was then knotted again, leaving a loop to attach the tendon to the force transducer. The mouse was then positioned on the platform, which was kept at 37° for the duration of the experiment. The leg limb was secured to the platform by putting a pin through the kneecap and taping down the foot. The loop of suture attached to the TA tendon was then attached to a 205B dual-mode servomotor transducer (Aurora Scientific, Aurora, ON, Canada). Finally, two electrical probes were placed in the biceps femoral muscle near the sciatic nerve for stimulation.

The resting force was set to between 3 and 4 *g* for a 10-min equilibrium period. The TA muscle was then stimulated at the optimal length (L_o,_ mm), and active tetanic muscle force was recorded to give the absolute force measurement using the Lab View-based DMC program (Aurora Scientific). The muscle was then evaluated using a 10-step passive stretch protocol, during which stimulation was applied to determine the force drop following repeated eccentric contractions. At each step, the TA muscle was passively strained 10% of the L_o_. Once the protocol was complete, the mice were given a lethal dose of ketamine/xylazine and the TA muscle was removed and weighed. The cross-sectional area measured as mass (g)/(muscle density × ratio of fiber length × L_o_), where muscle density is 1.06 mg/mm^3^ and the ratio of fiber length in the TA is 0.6.^21^ The cross-sectional area using the muscle weight and L_o_ was applied to the absolute force measurement to give the specific force measurement.

### Statistics

Statistical analysis was performed in GraphPad Prism (version 8.4 for Windows, GraphPad Software, San Diego, CA, USA). Absolute and specific force measurements were analyzed using a Kruskal-Wallis nonparametric test with Dunn’s multiple comparison post hoc test. Eccentric contraction force drop data were analyzed using two-way ANOVA with Holm-Sidak multiple comparison post hoc test. IF and immunoblot results were analyzed across doses using one-way ANOVA with a post hoc test for linear trend from left to right. For all analyses, p <0.05 was the threshold for significance. Datasets with n ≤ 3 were not statistically analyzed due to low statistical power. For functional data, the mean and standard deviation were determined for each measurement, and subsequently measurements that were more than ± 1 standard deviation were removed as outliers. The tenth recording for eccentric contractions was used to determine outliers.

## Supplemental information

Document S1. Figures S1–S3

Document S2. Article plus supplemental information
