# Supplementary material for: Pre-clinical dose-escalation studies establish a therapeutic range for U7snRNA-mediated DMD exon 2 skipping
Source: Mol Ther Methods Clin Dev. 2021 Mar 23;21:325–40. doi: 10.1016/j.omtm.2021.03.014 (PMC8047432; doi:10.1016/j.omtm.2021.03.014)
Supplement: Document S1. Figures S1–S3 [file mmc1.pdf]

OMTM, Volume *21*

Supplemental information

Pre-clinical dose-escalation studies establish a therapeutic range for U7snRNA-mediated *DMD* exon 2 skipping

Tabatha R. Simmons, Tatyana A. Vetter, Nianyuan Huang, Adeline Vulin-Chaffiol, Nicolas Wein, and Kevin M. Flanigan

Supplementary Material

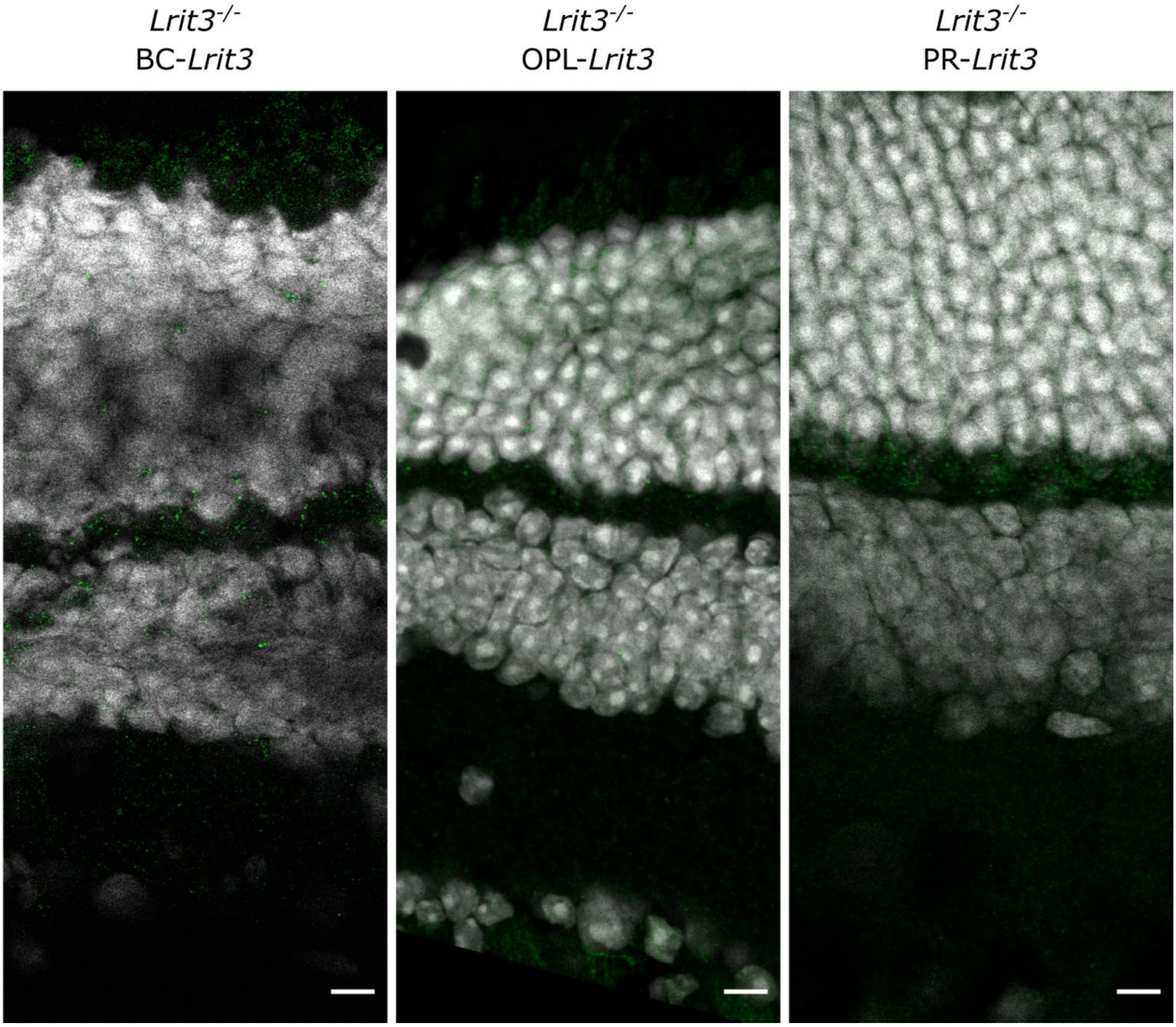

Figure S1: Localization of LRIT3

Representative confocal images of cross-sections of *Lrit3*$^{-/-}$-BC-*Lrit3*, *Lrit3*$^{-/-}$-OPL-*Lrit3* and *Lrit3*$^{-/-}$-PR-*Lrit3* retinas stained with an antibody against LRIT3 (green). Scale bar, 10 µm.

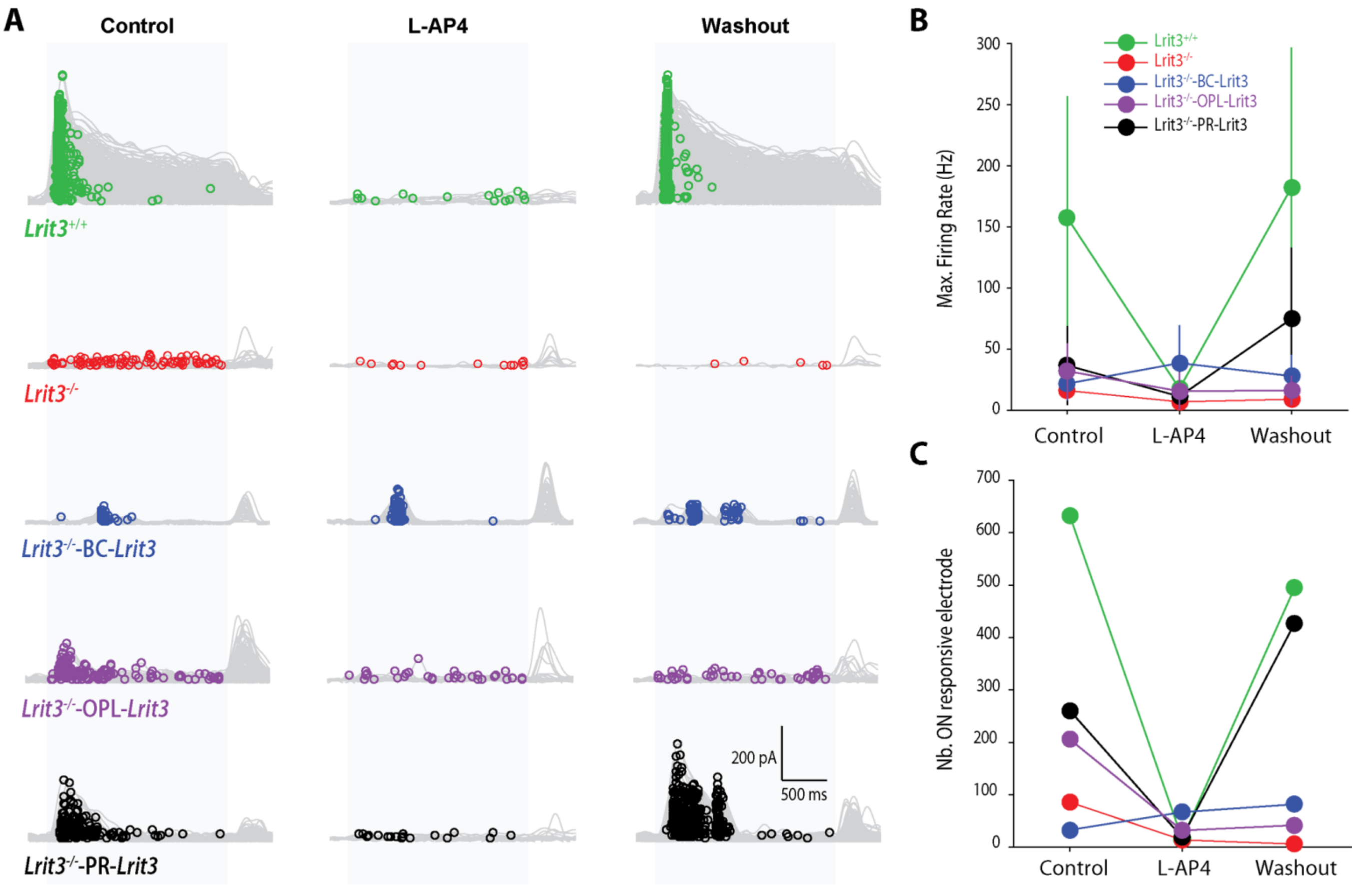

Figure S2: L-AP4 treatment reduce restored ON responses.

A) Spike density function for all recorded electrodes with ON responses (grey), before (control), during (L-AP4), and after (washout) perfusion of L-AP4. Peak indicated on individual traces during the light stimulation (light grey area). B) Peak firing rate for the different condition during the pharmacological experiment. C) Evolution of the number of electrodes with an ON response with the addition and removal of L-AP4 in the solution.

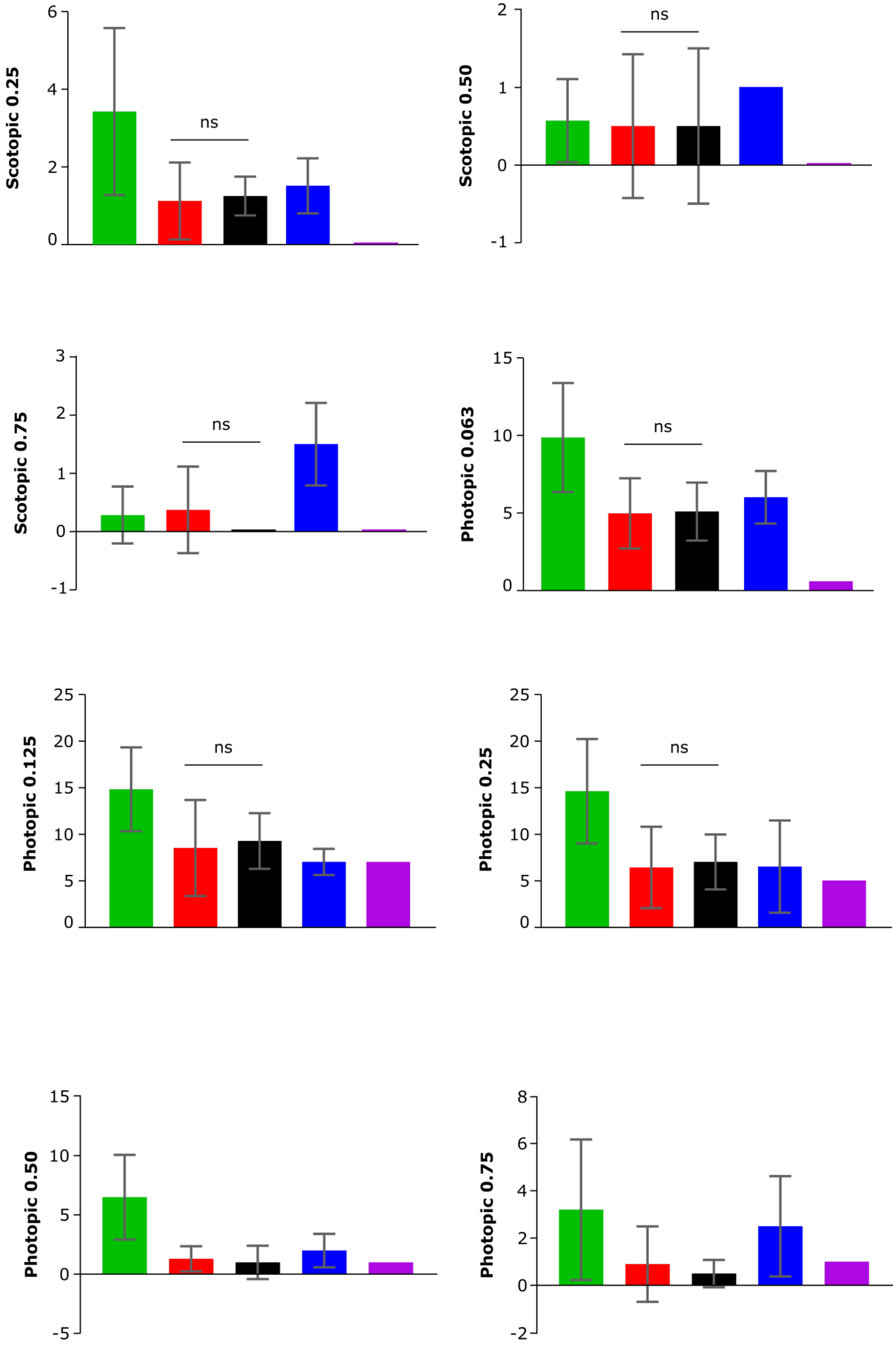
Scotopic 0.25
6
4
2
0
ns
Scotopic 0.50
2
1
0
-1
ns
Scotopic 0.75
3
2
1
0
-1
ns
Photopic 0.063
15
10
5
0
ns
Photopic 0.125
25
20
15
10
5
0
ns
Photopic 0.25
25
20
15
10
5
0
ns
Photopic 0.50
15
10
5
0
-5
Photopic 0.75
8
6
4
2
0
-2

Figure S3: Measurements of optomotor responses under both scotopic and photopic conditions

A) Histogram representation of the number of head movements per minute which was obtained under scotopic conditions with spatial frequencies from 0.25 to 0.75 cycles/degree for *Lrit3*$^{-/-}$-BC-*Lrit3* (blue), *Lrit3*$^{-/-}$-OPL-*Lrit3* (purple) and *Lrit3*$^{-/-}$-PR-*Lrit3* (black) mice and compared using Mann-Whitney statistical test with representative *Lrit3*$^{+/+}$ (green) and *Lrit3*$^{-/-}$ (red) mice. B) Histogram representation of the number of head movements per minute which was obtained under photopic conditions with spatial frequencies from 0.063 to 0.75 cycles/degree for *Lrit3*$^{-/-}$-BC-*Lrit3* (blue), *Lrit3*$^{-/-}$-OPL-*Lrit3* (purple) and *Lrit3*$^{-/-}$-PR-*Lrit3* (black) mice and compared using Mann-Whitney statistical test with representative *Lrit3*$^{+/+}$ (green) and *Lrit3*$^{-/-}$ (red) mice.
